# PINK1 Is Dispensable for Mitochondrial Recruitment of Parkin and Activation of Mitophagy in Cardiac Myocytes

**DOI:** 10.1371/journal.pone.0130707

**Published:** 2015-06-25

**Authors:** Dieter A. Kubli, Melissa Q. Cortez, Alexandra G. Moyzis, Rita H. Najor, Youngil Lee, Åsa B. Gustafsson

**Affiliations:** Skaggs School of Pharmacy and Pharmaceutical Sciences, University of California, San Diego, La Jolla, California, United States of America; Rutgers New Jersey Medical School, UNITED STATES

## Abstract

Myocyte function and survival relies on the maintenance of a healthy population of mitochondria. The PINK1/Parkin pathway plays an important role in clearing defective mitochondria via autophagy in cells. However, how the PINK1/Parkin pathway regulates mitochondrial quality control and whether it coordinates with other mitophagy pathways are still unclear. Therefore, the objective of this study was to investigate the effect of PINK1-deficiency on mitochondrial quality control in myocytes. Using PINK1-deficient (PINK1-/-) mice, we found that Parkin is recruited to damaged cardiac mitochondria in hearts after treatment with the mitochondrial uncoupler FCCP or after a myocardial infarction even in the absence of PINK1. Parkin recruitment to depolarized mitochondria correlates with increased ubiquitination of mitochondrial proteins and activation of mitophagy in PINK1-/- myocytes. In addition, induction of mitophagy by the atypical BH3-only protein BNIP3 is unaffected by lack of PINK1. Overall, these data suggest that Parkin recruitment to depolarized cardiac mitochondria and subsequent activation of mitophagy is independent of PINK1. Moreover, alternative mechanisms of Parkin activation and pathways of mitophagy remain functional in PINK1-/- myocytes and could compensate for the PINK1 deficiency.

## Introduction

Mitochondria play a critical role in myocytes by providing them with ATP via oxidative phosphorylation. Mitochondria are also important for other critical cellular processes including the sequestration of excess cytosolic calcium [1]. Myocyte function and survival therefore depends on the maintenance of a healthy population of mitochondria. A mitochondrion that is dysfunctional or damaged not only produces less ATP, but can also produce excess reactive oxygen species (ROS) and release proteins that activate cell death pathways [2]. Therefore, cells have mitochondrial quality control mechanisms in place to ensure a quick response to damage resulting from changes in the cellular environment. Protein-level quality control includes the removal of dysfunctional proteins by mitochondrial proteases [3] or through segregation into mitochondria derived vesicles [4]. If mitochondrial damage is overwhelming, it becomes necessary to remove the entire mitochondrion through degradation by macroautophagy, also known as mitochondrial autophagy or mitophagy. In this process, an autophagosome sequesters the dysfunctional mitochondrion and delivers it to the lysosome for degradation [5]. Failure to remove dysfunctional mitochondria can have severe consequences for the cell and may lead to activation of cell death.

The PINK1/Parkin pathway plays an important role in marking dysfunctional mitochondria for degradation. Parkin is localized to the cytosol under baseline conditions, but rapidly translocates to damaged mitochondria via a mechanism that is thought to involve the serine/threonine kinase PINK1 [6, 7]. Healthy mitochondria with intact membrane potential (∆Ψm) rapidly import and degrade PINK1 via mitochondrial proteases [7, 8]. Upon loss of membrane potential, import of PINK1 is halted and PINK1 accumulates on the outer mitochondrial membrane. This leads to the recruitment and activation of the E3 ubiquitin ligase Parkin [9–12]. Once Parkin is at the mitochondria, it ubiquitinates several different proteins on the outer mitochondrial membrane which then serve as signals for autophagic clearance [6, 13].

Mutations in the PINK1/Parkin pathway are associated with juvenile recessive forms of Parkinson’s disease and neuronal defects [14, 15]. Recent studies have also implicated Parkin as a regulator of mitochondrial degradation in cardiac myocytes. For instance, Parkin-deficient mice accumulate dysfunctional mitochondria in the myocardium with age, which correlates with increased oxidative damage and declining mitochondrial respiration [16, 17]. In addition, Parkin plays a critical role in the removal of dysfunctional mitochondria in myocytes in response to myocardial infarction [17]. Although these studies demonstrate an important role for Parkin-mediated mitochondrial clearance in the heart, the underlying mechanisms of mitochondrial recruitment and activation of Parkin in myocytes are still unclear. While several studies suggest that PINK1 function is required for Parkin translocation, there is evidence that Parkin can act independently of PINK1 in *Drosophila melanogaster* [18–20]. Mitophagy can also be mediated via autophagy receptors in the outer mitochondrial membrane including BNIP3, NIX, and FUNDC1 [21–24], and it is unknown whether these receptors can compensate for a defect in the PINK1/Parkin pathway.

In this study, we have investigated whether PINK1 deficiency affects mitochondrial clearance in cardiac myocytes. We report that both Parkin translocation to damaged cardiac mitochondria and activation of mitophagy occur independently of PINK1. In addition, BNIP3-mediated mitophagy is also functional in PINK1-/- myocytes. These studies demonstrate the presence of multiple mechanisms of Parkin activation, as well as alternative mitophagy pathways, in myocytes that can compensate for impaired PINK1 signaling.

## Materials and Methods

### Animals

All animal protocols were in accordance with institutional guidelines and approved by the Institutional Animal Care and Use Committee of the University of California San Diego. *Park2* knockout mice were obtained from Jackson Laboratories (B6.129S4-*Park2* ^*tm1Shn*^/J, stock number 006582) and have been described previously [17, 25]. *PINK1* knockout mice were also obtained from Jackson Laboratories (B6;129-*Pink1* ^*tm1Aub*^/J, stock number 013050 and have been described previously [26]. Myocardial infarctions were performed as described previously [17]. Four hours post-MI, infarct border zone tissue was dissected under a dissecting microscope for mitochondria isolation and western blotting as described below.

### FCCP Treatments

Hearts were rapidly excised, placed in Krebs-Henseleit (KH) perfusion buffer and cannulated through the aorta. Retrograde perfusion was performed with KH perfusion buffer for 5 minutes for equilibration and stabilization followed by perfusion with 100 nM FCCP or DMSO in perfusion buffer for up to 15 minutes. At the indicated timepoints, hearts were cut down and ventricles were rinsed in mitochondria isolation buffer. Mitochondria were isolated immediately following perfusion. For treatment of mice with the mitochondrial uncoupler FCCP, mice were administered 1 mg per kg body weight of FCCP in saline twice daily by intraperitoneal injection. The final dose of FCCP was given 1 hour prior to collecting tissue.

### Mitochondria Isolation

Mitochondria were isolated as previously described [17]. Heart tissue was homogenized in ice-cold isolation buffer containing 10 mM MOPS pH 7.4, 250 mM sucrose, 5 mM KH_2_PO_4_, 2 mM MgCl_2_, 1 mM EGTA, and 0.1% fatty acid-free BSA. The final mitochondrial pellet was lysed in RIPA buffer (50 mM Tris-HCl pH 8.0, 150 mM NaCl, 1% Triton X-100, 0.5% sodium deoxycholate, 0.1% SDS, 1 mM EGTA, 1 mM EDTA, and Complete protease inhibitor (Roche Applied Bioscience) for western blotting.

### Western Blotting

Heart lysates were prepared as previously described in lysis buffer comprised of 50 mM Tris-HCl (pH 7.4), 150 mM NaCl, 1 mM EGTA, 1 mM EDTA, 1% Triton X-100, and Complete protease inhibitor cocktail (Roche Applied Bioscience) [17]. To prepare cardiac myocyte lysates, cells were scraped off dishes in cold PBS and centrifuged at 200 × g at 4˚C for 5 minutes to collect cell pellets. Pellets were resuspended in RIPA buffer and passed through a 27G needle five times to shear cells. Lysates were incubated on ice for 30 minutes and then cleared by centrifugation at 20 000 × g for 20 minutes. Proteins in the supernatants were separated by SDS-PAGE, transferred to nitrocellulose, and immunoblotted with antibodies directed against: Parkin (Cell Signaling 4211, 1:1000); PINK1 (Cayman Chemical 10006283, 1:1000); Complex III Core 2 (Invitrogen 459220, 1:1000); Complex IV subunit 1 (Invitrogen 459600, 1:1000); LC3A/B (Cell Signaling 4108, 1:1000), Ubiquitin (Santa Cruz sc-8017, 1:250), TOM20 (Santa Cruz sc-11415, 1:1000), and GAPDH (Genetex 627408, 1:1000).

### Adult Mouse Myocyte Isolation

Adult mouse cardiac myocytes were isolated as previously described [22]. Cardiac myocytes were plated on 35 mm glass bottom dishes (MatTek) at a density of 100,000 cells per dish in a volume of 2 mL plating medium. Cells were allowed to plate down for 2 hours before infection with GFP-LC3, mCherry-Parkin, Mito-GFP, BNIP3, BNIP3ΔTM, or β-Gal adenoviruses at 100 MOI. Cells were incubated with adenoviruses for 3 hours, rinsed with plating medium, and then allowed to recover in plating medium for 18 hours before treatment.

### Fluorescence Microscopy

Myocytes were treated with DMSO or 40 μM rotenone in plating medium. For imaging, cells were fixed at the specified timepoints in 4% paraformaldehyde, permeabilized in either 0.2% Triton X-100 in PBS or 0.2% saponin for LAMP2 staining, for 30 minutes at 37°C. Blocking and antibody applications were performed in 5% normal goat serum + 0.2% Triton X-100 in PBS. Antibodies used included TOM20 (Santa Cruz, 1:100), and Complex IV subunit 1 (Invitrogen, 1:1000), and LAMP2 (Abcam ab13524, 1:100). Following antibody labeling, Hoechst 33342 (Life Technologies) was applied to label nuclei. Cells were imaged by fluorescence microscopy on a Carl Zeiss AxioObserver Z1 equipped with a motorized Z-stage and an ApoTome for optical sectioning. Z-stacks were acquired in ApoTome mode using a high-resolution AxioCam MRm digital camera, a 63X Plan-Apochromat oil-immersion objective and Zeiss AxioVision 4.8 software (Carl Zeiss).

### Electron Microscopy

Adult mouse cardiac myocytes were isolated as described above and plated on 60-mm dishes at a density of 250,000 cells. Medium was changed 2 hours after plating, and cells were allowed to recover overnight. The following day, cells were treated with 40 μM rotenone or DMSO and then fixed with 2% glutaraldehyde in 0.1 M sodium cacodylate buffer pH 7.4. Cells were collected by scraping dishes followed by centrifugation. Cell pellets were post fixed with 1% osmium tetroxide in 0.1 M cacodylate buffer, stained with 2% uranyl acetate, and then dehydrated in ethanol. Samples were embedded in Durcupan embedding medium (Sigma-Aldrich). Sections of 60 nm thickness were imaged on Formvar and carbon-coated copper grids. Digital images were obtained on a Tecnai G2 Spirit by FEI equipped with an Eagle 4k HS digital camera. Sample preparation and imaging was performed at the UCSD Electron Microscopy Facility under the direction of Dr. Marilyn Farquhar.

### Statistical Analyses

All values are expressed as Mean ± standard error of mean (SEM). Statistical analyses were performed using Student’s t-test or ANOVA followed by Tukey’s Multiple Comparison test.

## Results

### PINK1 Is Dispensable for Parkin Translocation to Cardiac Mitochondria

**Fig 1:**
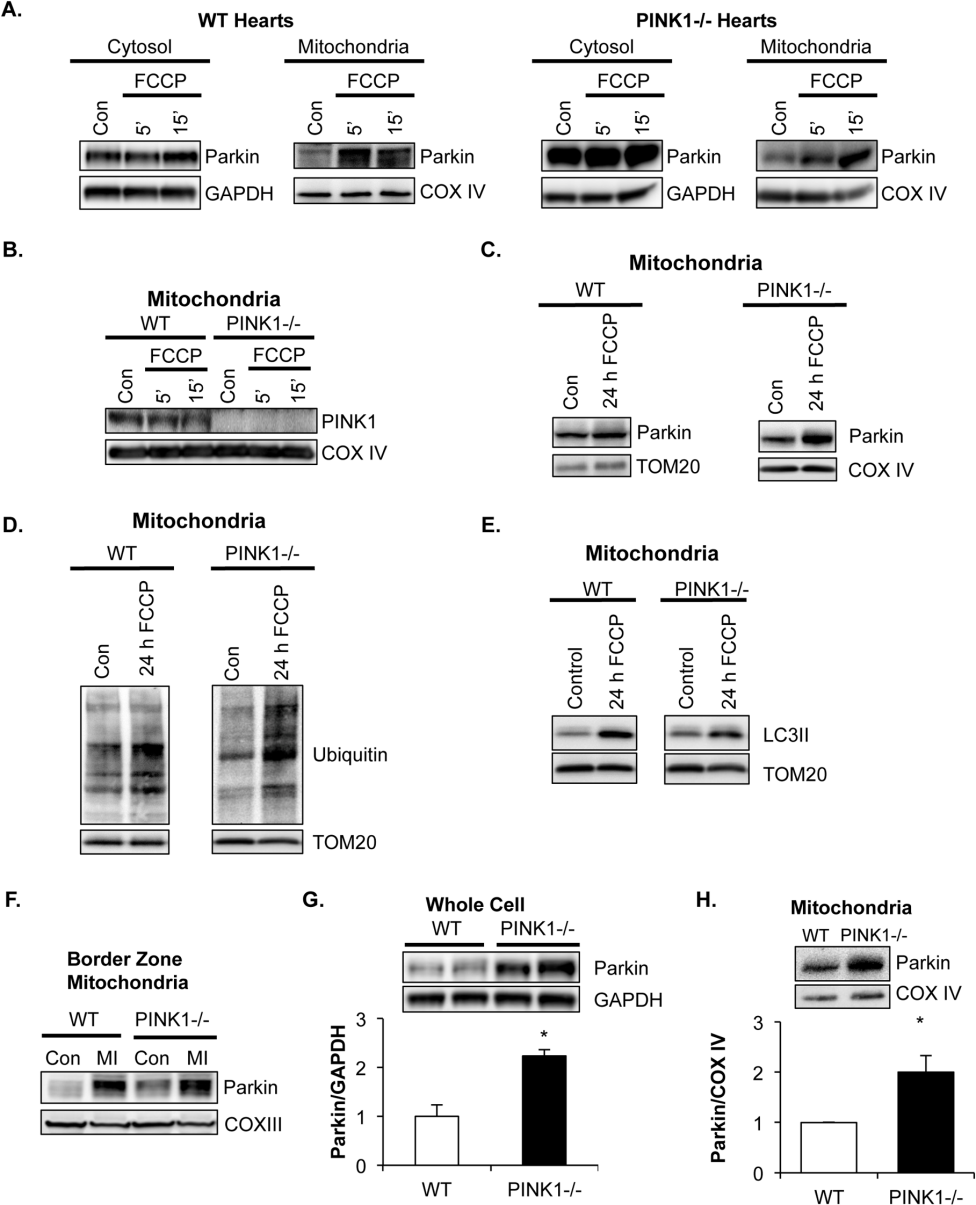
Translocation of Parkin to mitochondria and activation of mitophagy occur independently of PINK1. (A) Perfusion of WT and PINK1-/- hearts with the mitochondrial uncoupler FCCP (100 nM) led to accumulation of Parkin in the mitochondrial fraction within 15 minutes (15’). (B) Perfusion of WT hearts with FCCP for up to 15 minutes did not result in accumulation of PINK1 in the mitochondrial fraction. PINK1-/- perfused heart samples shown for comparison. In vivo FCCP treatment for 24 h led to (C) translocation of Parkin to cardiac mitochondria, (D) increased ubiquitination of cardiac mitochondrial proteins, and (E) increased LC3II association with mitochondria. (F) Analysis of mitochondria isolated from the infarct border zone four hours after *in vivo* myocardial infarction (MI). Parkin levels increased in the mitochondrial fraction in both WT and PINK1-/- hearts after MI. Representative western blots and densitometry data show significantly elevated Parkin protein levels in the heart (G) and at the mitochondria (H) in 3 month old PINK1-/- mice. Mean ± SEM, n = 3, *p<0.05 vs. WT.

While most studies have reported that PINK1 is a critical upstream regulator of Parkin, a few have found that Parkin can function independently of PINK1 in *Drosophila* [18–20]. To investigate the relationship between PINK1 and Parkin in the heart, we perfused hearts from WT and PINK1-deficient mice with the mitochondrial uncouple FCCP to activate Parkin-mediated mitophagy [27]. Surprisingly, we found that Parkin levels rapidly increased in the mitochondrial fraction in both WT and PINK1-/- hearts after perfusion with FCCP (Fig 1A), suggesting that PINK1 is not required for the recruitment of Parkin to depolarized cardiac mitochondria. Studies have suggested that the accumulation of PINK1 triggers recruitment of Parkin to mitochondria [6, 28, 29], but we found that perfusion of WT hearts with FCCP for 5 or 15 min did not result in accumulation of endogenous PINK1 at the mitochondria (Fig 1B). This suggests that stabilization of PINK1 does not occur within this short timeframe and that recruitment of Parkin can occur prior to PINK1 accumulation in the myocardium. Although we observed rapid translocation of Parkin to mitochondria in both WT and PINK1-/- in response to FCCP perfusion, we did not observe an increase in ubiquitination of mitochondrial proteins at these time points, suggesting that Parkin was not yet activated (data not shown).

We next investigated whether Parkin translocation to uncoupled mitochondria occurred in PINK1-/- mice *in vivo*. We injected WT and PINK1-/- mice with FCCP to activate mitophagy in the myocardium [16]. We found that Parkin translocated to mitochondria in both WT and PINK1-/- hearts after initiating the FCCP treatment (Fig 1C). Parkin accumulation on mitochondria correlated with increased ubiquitination of mitochondrial proteins (Fig 1D) and increased association of the autophagy protein LC3II with the mitochondria after FCCP treatment (Fig 1E). These data suggest that Parkin-mediated mitophagy is still activated in the absence of PINK1 in response to mitochondrial damage.

To confirm that PINK1 is not required for recruitment of Parkin to mitochondria *in vivo*, we subjected WT and PINK1-/- mice to myocardial infarction (MI). We previously reported that Parkin is recruited to mitochondria in the infarct border zone 4 hours post-MI [17]. We confirmed that four hours of MI was sufficient to induce translocation of Parkin to mitochondria in the infarct border zone of WT mice. Parkin also translocated to PINK1-/- mitochondria after 4 hours of MI, confirming that PINK1 is not necessary for recruitment of Parkin (Fig 1F). In addition, we observed that hearts from untreated PINK1-/- mice contained significantly more total Parkin protein than WT hearts (Fig 1G) and that more Parkin localized to PINK1-/- mitochondria at baseline (Fig 1H). This suggests that Parkin protein levels might be elevated to compensate for the lack of PINK1.

**Fig 2:**
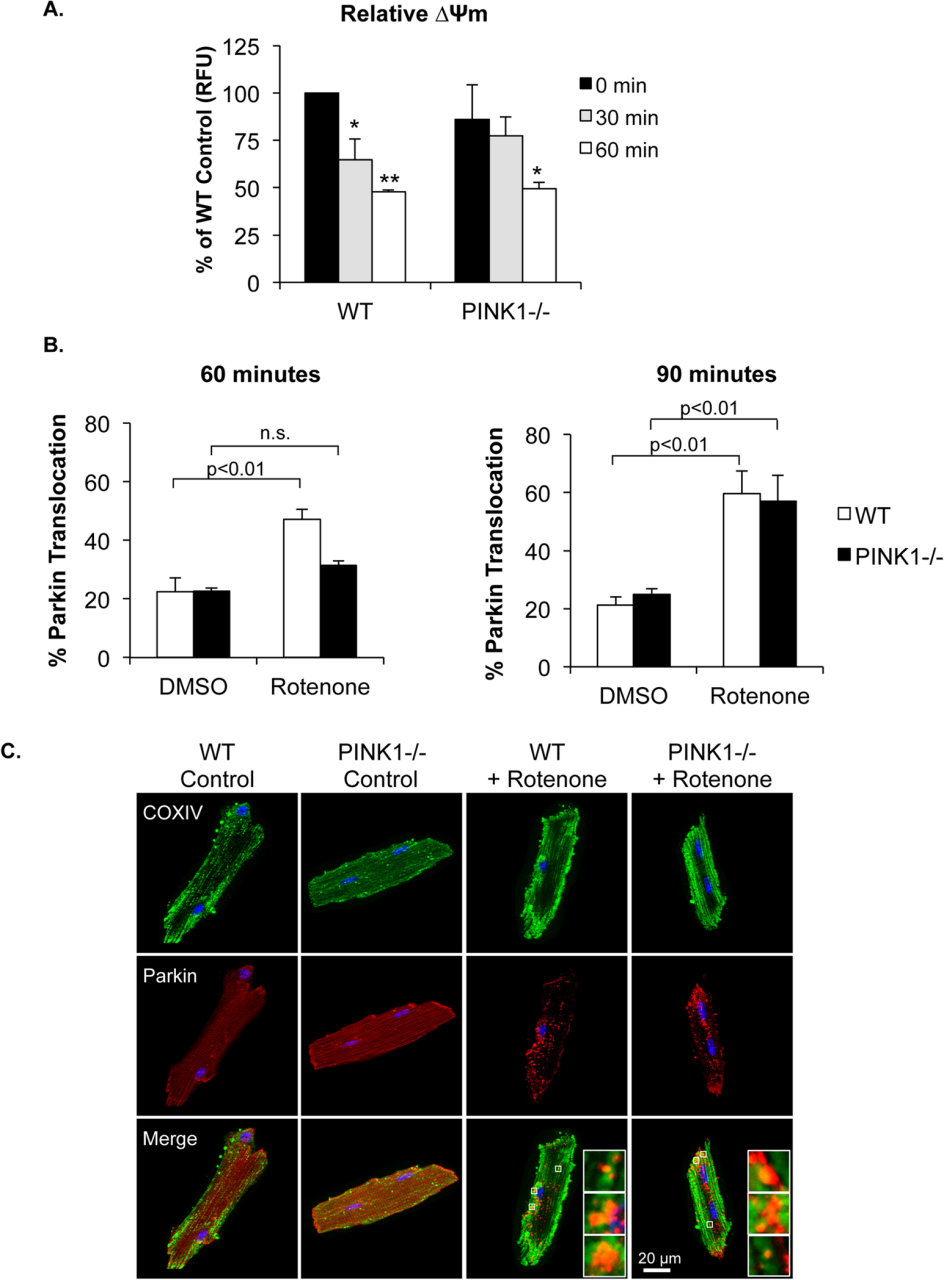
Rotenone induces delayed Parkin translocation to mitochondria in PINK1-/- myocytes. (A) The mitochondrial complex I inhibitor rotenone (40 μM) significantly reduced mitochondrial membrane potential by 1 hour of treatment in WT and PINK1-/- cardiac myocytes (n = 3). (B) Quantitation of percentage of cells with Parkin translocation to mitochondria after 60 and 90 min of rotenone treatment. (C) Representative images. Mean ± SEM, n = 3, *p<0.05, **p<0.01 vs. 0 min. n.s. = not significant

To further verify that PINK1 is dispensable for Parkin recruitment, we assessed whether Parkin translocated to mitochondria in PINK1-/- cardiac myocytes treated with rotenone. Rotenone is a mitochondrial complex I inhibitor and exposure of myocytes to rotenone resulted in a significant decrease in ∆Ψm between 30 and 60 minutes of treatment (Fig 2A). We found that 60 minutes of rotenone treatment resulted in translocation of mCherry-Parkin to mitochondria in a significant number of WT myocytes (Fig 2B and 2C). Interestingly, significant Parkin translocation to mitochondria was not observed until 90 min of rotenone treatment in PINK1-/- cells (Fig 2B), suggesting that Parkin recruitment is delayed in PINK1-/- myocytes. These studies demonstrate that Parkin retains its ability to translocate to dysfunctional mitochondria in PINK1-/- cardiac myocytes.

### Parkin Induces Mitophagy in PINK1-/- Myocytes

**Fig 3:**
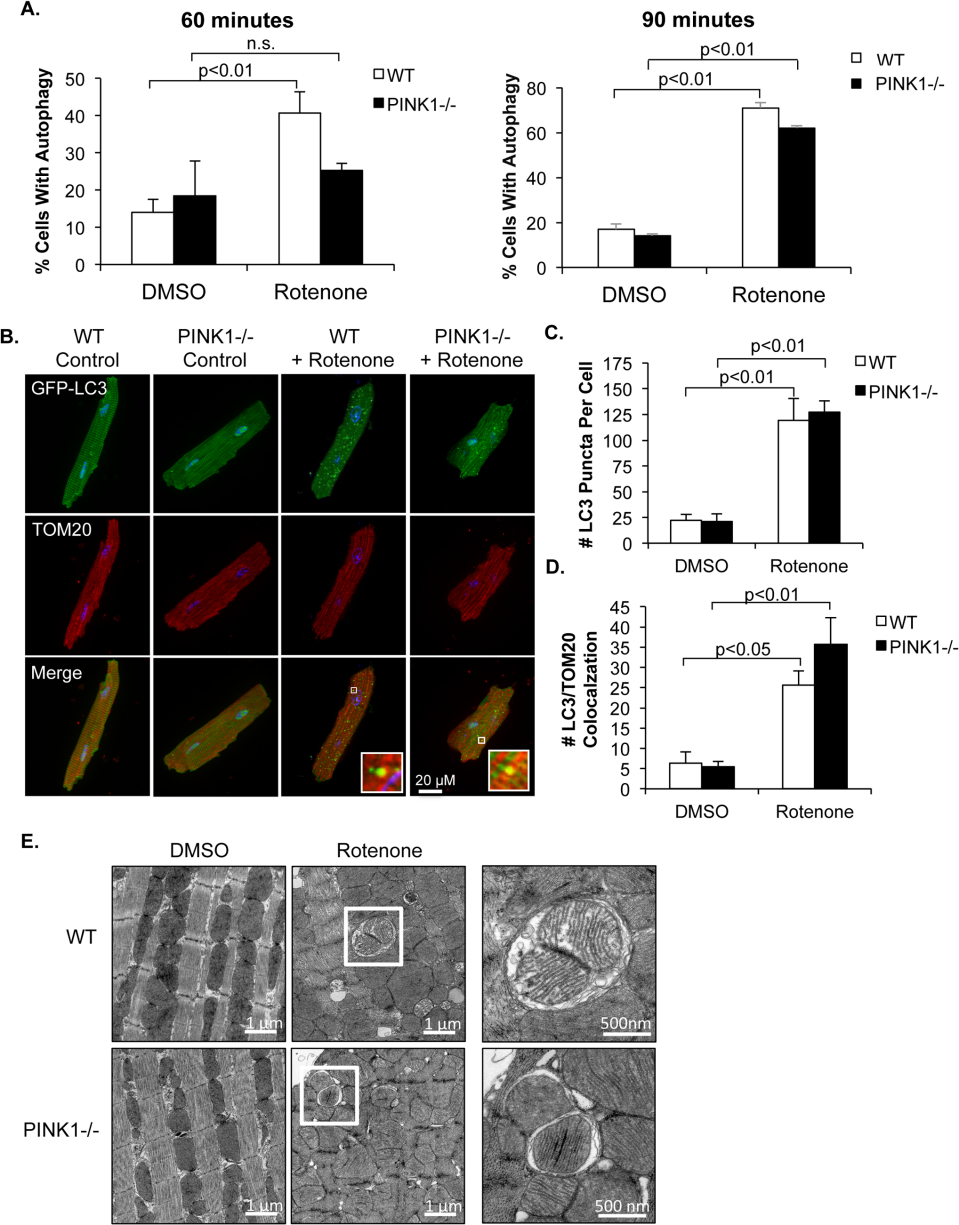
Rotenone induces activation of autophagy and mitophagy in WT and PINK1-/- adult mouse myocytes. (A) Quantitation of autophagy activation after 60 and 90 min rotenone (40 μM) treatment in WT and PINK1-/- myocytes. (B) Representative fluorescent images of WT and PINK1-/- myocytes overexpressing GFP-LC3 and stained with anti-TOM20 to label mitochondria. Co-localization between GFP-LC3 and TOM20 indicate activation of mitophagy. (C) Quantitation of the number of GFP-LC3 puncta in cells. (D) Quantitation of the number of GFP-LC3 autophagosomes co-localizing with TOM20-labeled mitochondria. (E) Transmission electron microscopy of isolated adult mouse myocytes after treatment with DMSO or 40 μM rotenone. Autophagosomes containing mitochondria were identified in both WT and PINK1-/- cardiac myocytes after rotenone treatment. Mean ± SEM, n = 3. n.s. = not significant

Next, we investigated whether Parkin promoted mitophagy in the absence of PINK1 in isolated myocytes. We first confirmed that rotenone treatment results in activation of autophagy in WT and PINK1-/- myocytes. Consistent with previous results [17], 60 min of rotenone treatment caused activation of autophagy in a significant number of WT myocytes (Fig 3A). Similar to the Parkin translocation experiments, we found that the activation of autophagy was delayed in PINK1-/- myocytes after rotenone treatment and only significantly activated after 90 min of rotenone treatment. When the number of autophagosomes per cell was quantified, we found that the average number of autophagosomes did not differ between WT and PINK1-/- cells (Fig 3B and 3C). Next, we assessed whether mitophagy was induced in rotenone-treated PINK1-/- myocytes with activated autophagy. We found a significant increase in the number of GFP-LC3 positive autophagosomes that co-localized with TOM20-labeled mitochondria in both WT and PINK1-/- myocytes after treatment with rotenone (Fig 3B and 3D). To confirm the presence of autophagosomes containing mitochondria following rotenone treatment, we examined isolated cardiac myocytes by transmission electron microscopy. Under baseline conditions, both WT and PINK1-/- myocytes had healthy mitochondria with dense cristae and a very low occurrence of autophagy (Fig 3E). Treatment with rotenone resulted in widespread mitochondrial swelling in both WT and PINK1-/- cells. Moreover, in accordance with our results from the fluorescence microscopy experiments, we observed autophagosomes containing mitochondria in both WT and PINK1-/- myocytes after rotenone treatment. Taken together, these data demonstrate that mitophagy is activated in both WT and PINK1-/- cardiac myocytes in response to mitochondrial damage.

**Fig 4:**
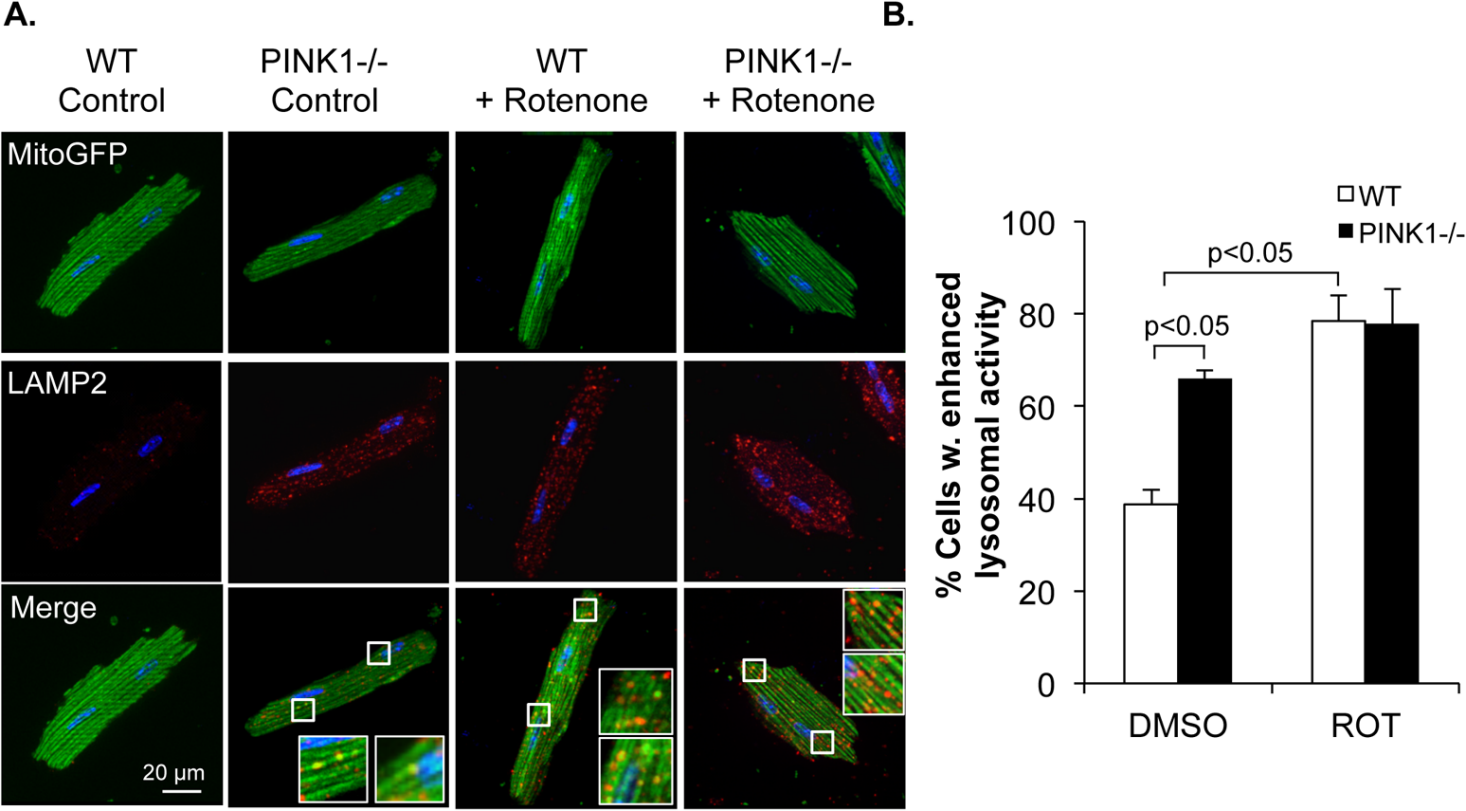
Autophagic flux is intact in rotenone treated PINK1-/- myocytes. (A) Representative images of WT and PINK1-/- myocytes overexpressing MitoGFP and stained with anti-LAMP2 after 90 min of rotenone treatment. (B) Quantitation of WT and PINK1-/- myocytes with enhanced lysosomal activity. Mean ± SEM, n = 3.

Once an autophagosome has sequestered its cargo, it fuses with a lysosome where the content is degraded [30]. We next confirmed that the autophagosomes containing mitochondria were delivered to lysosomes in both WT and PINK1-/- myocytes after rotenone treatment. We found that the number of lysosomes were significantly increased in WT cells after rotenone treatment (Fig 4A and 4B). Surprisingly, PINK1-/- myocytes already had a significant increase in lysosomes at baseline compared to WT cells (Fig 4A and 4B). We also confirmed that many MitoGFP labeled mitochondria colocalized with LAMP2-positive lysosomes in both WT and PINK1-/- myocytes after rotenone treatment (Fig 4A). Overall, these results confirm that PINK1-/- cardiac myocytes are capable of degrading dysfunctional mitochondria via the autophagosomal-lysosomal pathway.

### Receptor-Mediated Mitophagy is Independent of PINK1

**Fig 5:**
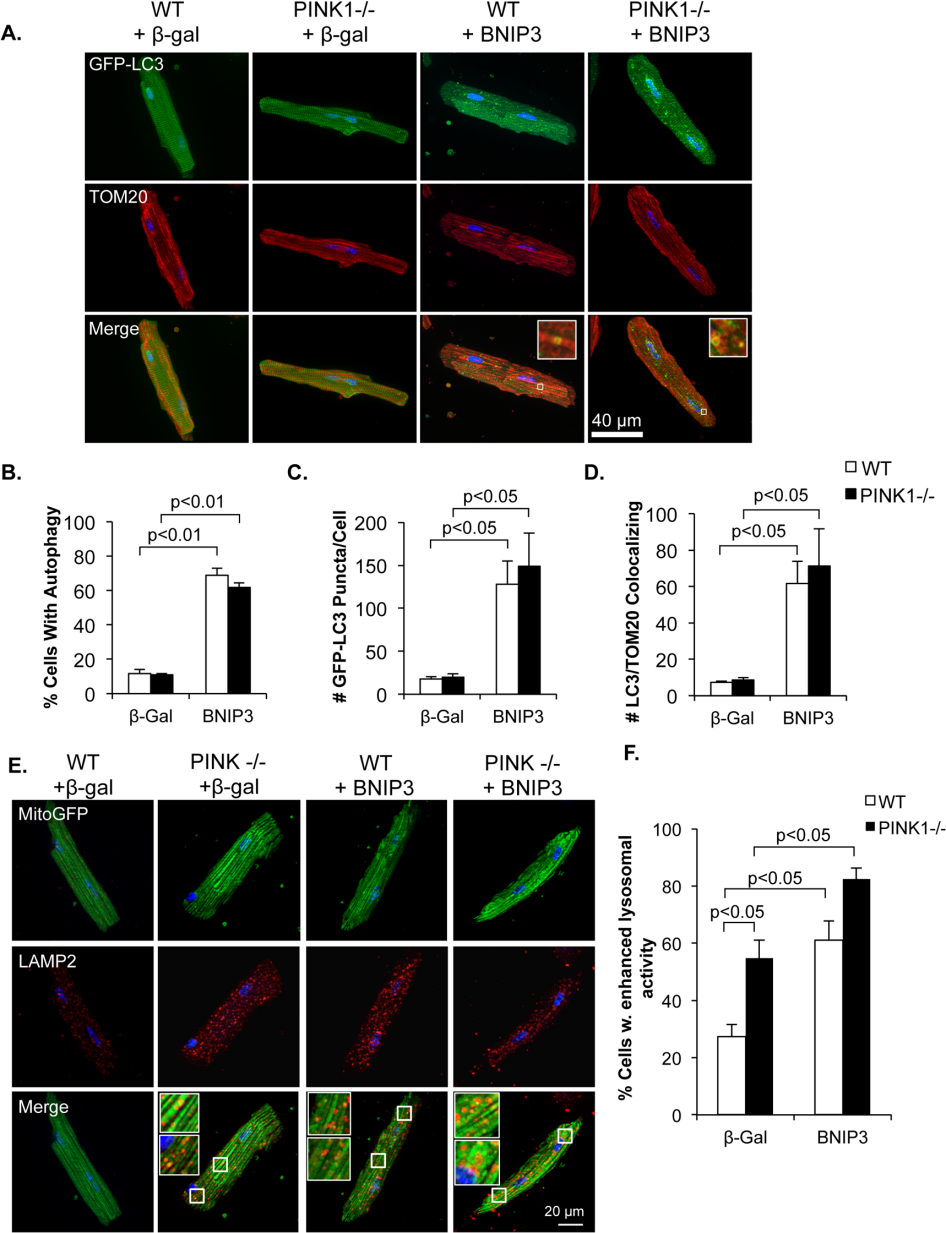
BNIP3 induces mitophagy in WT and PINK1-/- myocytes. (A) Representative fluorescent images of WT and PINK1-/- adult mouse cardiac myocytes overexpressing GFP-LC3 and either β-gal or BNIP3. (B) The percentage of cells with activated autophagy significantly increased in both WT and PINK1-/- cells in response to BNIP3 overexpression. (C) Quantitation of GFP-LC3 puncta in adult mouse cardiac myocytes show no difference in autophagosome number in PINK1-/- cells compared to WT. (D) Quantitation of GFP-LC3 puncta colocalization with mitochondrial TOM20 show increased mitophagy in both WT and PINK1-/- myocytes in response to BNIP3. (E) Representative images of LAMP2 stained WT and PINK1-/- myocytes overexpressing β-gal or BNIP3. (F) Quantitation of WT and PINK1-/- myocytes with enhanced lysosomal activity. Mean ± SEM, n = 3–4.

Our data demonstrate that mitophagy still occurs in PINK1-/- myocytes after treatment with rotenone. However, it is possible that other mitophagy pathways may also be activated to compensate for the lack of PINK1. Therefore, we investigated whether alternative mechanisms of mitophagy remain functional in PINK1-/- cells. BNIP3 is a mitochondrial protein that can promote mitophagy by direct interaction with LC3 on the autophagosome via its LC3-interacting region [31]. To investigate if BNIP3 could induce autophagy and mitophagy in PINK1-/- myocytes, WT and PINK1-/- adult myocytes were infected with GFP-LC3 and either control or BNIP3 adenoviruses, and then fixed and stained for the mitochondrial marker TOM20. We found that autophagy was activated to the same extent in PINK1-/- as in WT cells in response to BNIP3 (Fig 5A–5C). Quantitation of the number GFP-LC3 positive autophagosomes co-localizing with TOM20-labeled mitochondria demonstrated that BNIP3-induced mitophagy is unaffected in PINK1-/- myocytes (Fig 5D). We also found that BNIP3 caused a significant increase in the number of lysosomes in both WT and PINK1-/- myocytes and that LAMP2-positive lysosomes colocalized with MitoGFP-labeled mitochondria in cells overexpressing BNIP3 (Fig 5E and 5F). Thus, BNIP3-mediated mitochondrial clearance via the autophagy-lysosomal pathway remains functional in the absence of PINK1.

**Fig 6:**
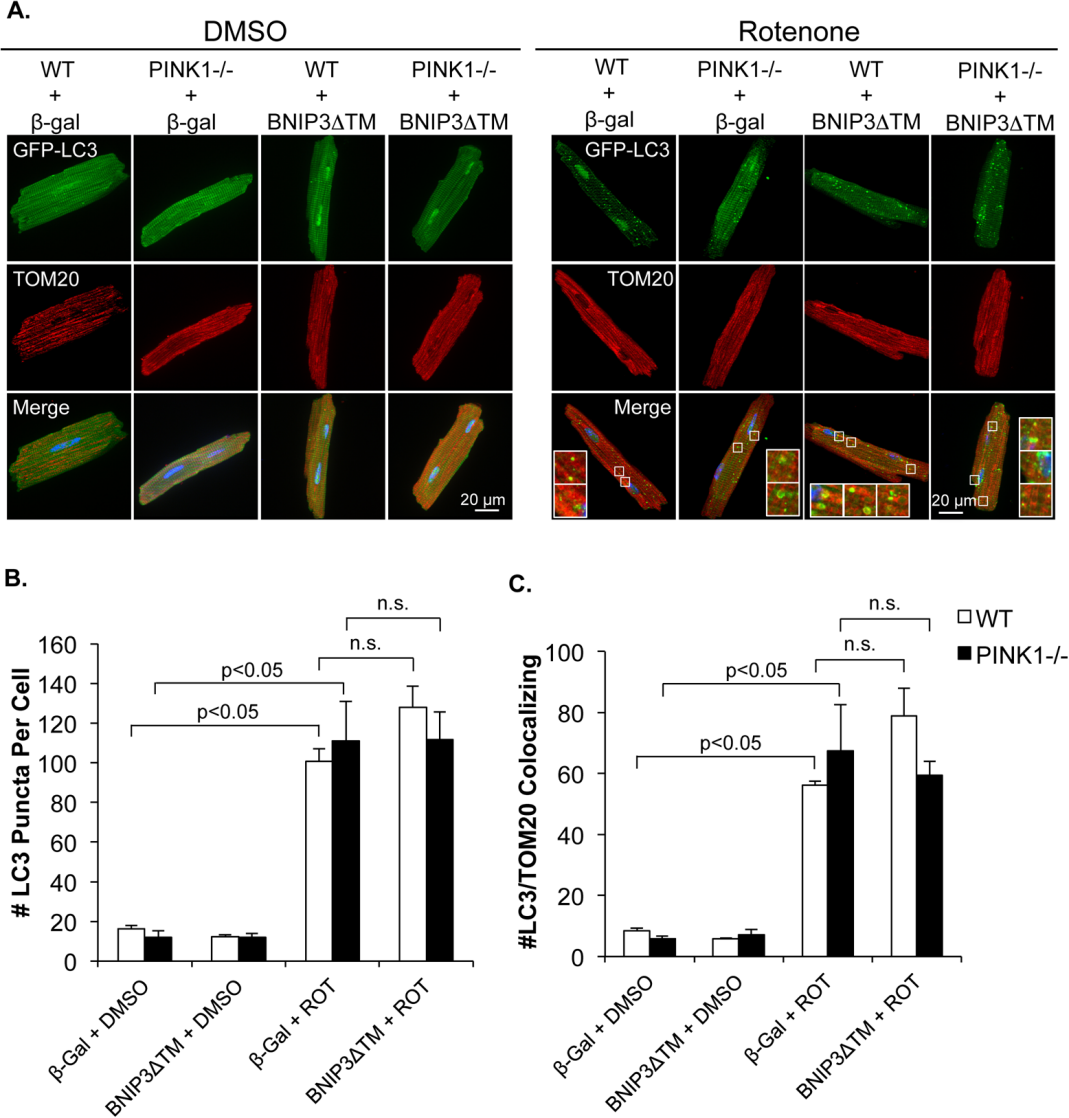
The dominant negative BNIP3ΔTM has no affect rotenone-mediated mitophagy. (A) Representative fluorescent images of WT and PINK1-/- adult mouse cardiac myocytes overexpressing β-gal or BNIP3ΔTM plus GFP-LC3. (B) Quantitation of the number of autophagosomes in WT and PINK1-/- myocytes. (C) Quantitation of GFP-LC3 puncta colocalization with mitochondrial TOM20. BNIP3ΔTM did not reduce rotenone-mediated mitophagy in either WT or PINK1-/- myocytes. Mean ± SEM, n = 3. n.s. = not significant

**Fig 7:**
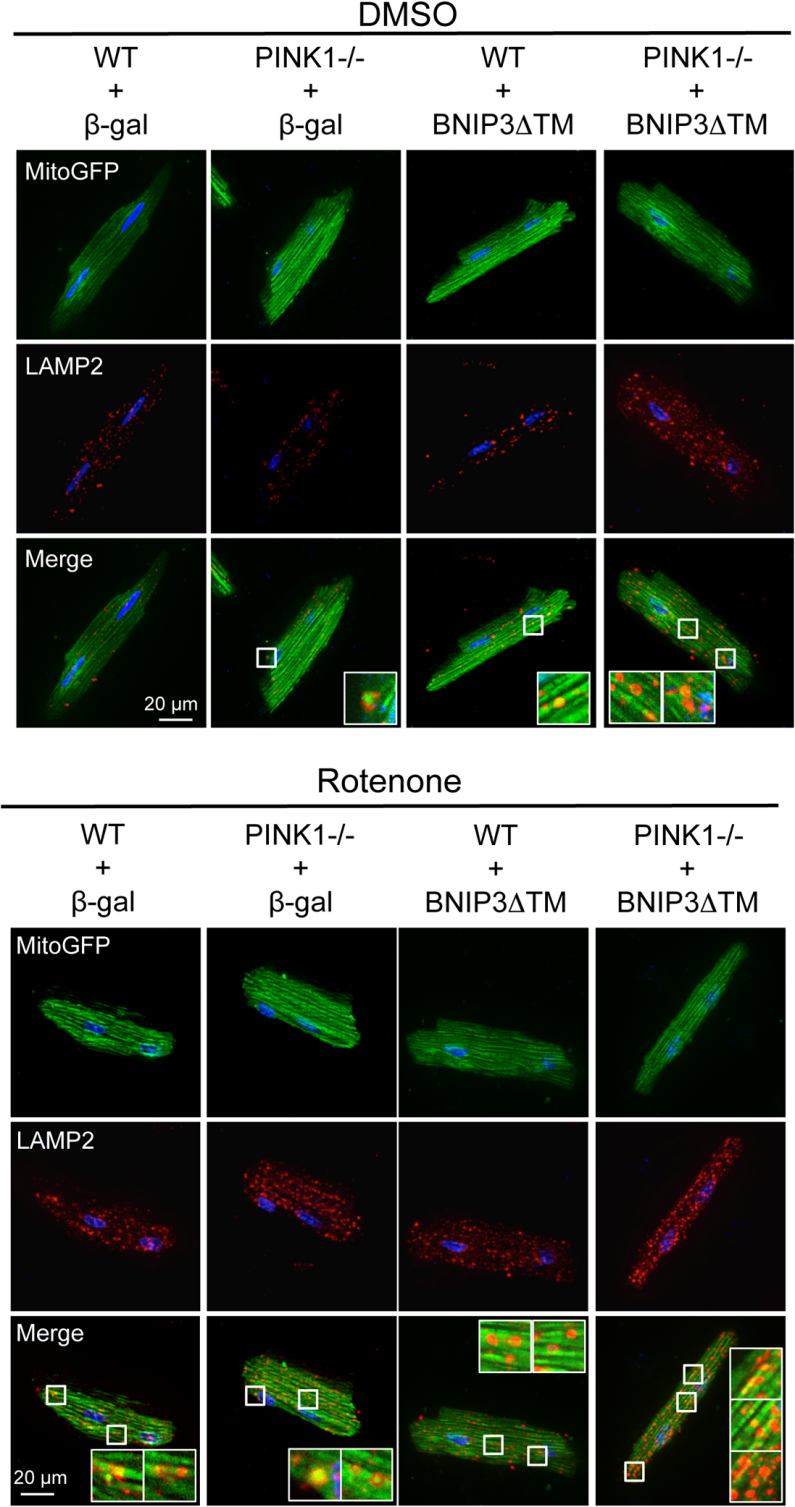
Bnip3ΔTM does not affect rotenone-stimulated autophagic flux. Representative images of LAMP2 stained WT and PINK1-/- myocytes overexpressing β-gal or BNIP3ΔTM plus MitoGFP after rotenone treatment (90 min).

To determine if BNIP3 contributes to activation of mitophagy in rotenone treated myocytes, we investigated whether a dominant negative mutant of BNIP3 that lacks the transmembrane domain (BNIP3ΔTM) [32] had an effect on rotenone-mediated mitophagy. However, we found that overexpression of BNIP3ΔTM in isolated WT and PINK1-/- cardiomyocytes had no effect on the number of autophagosomes in either WT or PINK1-/- cells after rotenone treatment (Fig 6A and 6B). Additionally, we found no reduction in the number of mitochondria co-localizing with GFP-LC3 positive autophagosomes in either WT or PINK1-/- myocytes overexpressing BNIP3ΔTM (Fig 6C), suggesting that BNIP3ΔTM did not affect rotenone-mediated mitophagy. We also confirmed that BNIP3ΔTM did not affect the delivery of mitochondria to lysosomes in rotenone-treated myocytes (Fig 7). This suggests that while BNIP3-mediated mitophagy is intact in PINK1-/- myocytes, it does not contribute to rotenone-mediated mitophagy.

## Discussion

In this study, we present several novel findings that bring new insights into mitochondrial quality control in cardiac myocytes. First, we discovered that mitochondrial translocation of Parkin and subsequent activation of mitophagy occur independently of PINK1 in myocytes. Our data also indicate that activation of Parkin-mediated mitophagy is delayed in the absence of PINK1. Finally, we confirmed that an alternative pathway of mitophagy remains fully functional in PINK1-/- myocytes. Thus, these findings suggest that multiple redundant mechanisms of quality control exist in myocytes to combat mitochondrial damage.

An important new finding in our study is that PINK1 is dispensable for mitochondrial translocation and activation of Parkin in cardiac myocytes. This is unexpected since most studies have reported that accumulation of PINK1 on the outer mitochondrial membrane is required for recruitment of Parkin [6, 7, 28, 29]. To the best of our knowledge, this is the first time that Parkin translocation and activation have been shown to function independently of PINK1 in a mammalian cell system. Although the activation of Parkin requires PINK1-mediated phosphorylation of both Mfn-2 [12] and ubiquitin [9–11], it is possible that other kinases can compensate for the lack of PINK1 in cell. Recent studies have reported that Parkin ubiquitinates several non-mitochondrial substrates, such as PARIS [33] and CD36 [34]. Since PINK1 is primarily a mitochondrial kinase, it is likely that another kinase is involved in Parkin-mediated activation and ubiquitination of non-mitochondrial substrates. The delay that we observed in Parkin translocation and activation in PINK1-/- myocytes could be due to different kinetics of activation and subcellular localization of this unknown kinase. Phosphorylation of ubiquitin at different residues has been documented in previous studies [35], but PINK1 is the only kinase to date that has been identified to phosphorylate ubiquitin. Other ubiquitin kinases remain elusive and future studies need to focus on identifying these kinases.

Moreover, our finding that Parkin protein levels are elevated in PINK1-deficient mouse hearts suggests that the cells are attempting to compensate for the PINK1 deficiency by increasing expression of Parkin. We also found elevated basal levels of Parkin at the mitochondria in PINK1-/- hearts, which further supports this hypothesis. Overexpression of Parkin has previously been reported to counteract PINK1 deficiency. For instance, PINK1 deficiency in *Drosophila melanogaster* leads to mitochondrial dysfunction, as well as muscle and neuronal degeneration, and this phenotype is rescued by transgenic overexpression of Parkin [18]. Similarly, overexpression of Parkin rescues mitochondrial abnormalities and reduces cell death in SH-SY5Y cells with stable knockdown of PINK1 [36]. High Parkin levels on the mitochondria of untreated PINK1-/- hearts may be indicative of elevated baseline mitochondrial stress, or may be a compensatory mechanism to prime the mitochondria for mitophagy in the absence of PINK1.

Our findings also demonstrate that the induction of autophagy and mitophagy by the atypical BH3-only protein BNIP3 is still intact in PINK1 deficient myocytes. Interestingly, inhibiting BNIP3 had no effect on rotenone-mediated mitophagy in either WT or PINK1-/- myocytes, indicating that BNIP3 is not compensating for the lack of PINK1 to mediate clearance of mitochondria under these conditions. It also confirms the existence of two different mitophagy pathways in cells. BNIP3 is a mitophagy receptor that degrades mitochondria by directly binding to LC3 on the autophagosome [31]. It also directly induces autophagy by disrupting the interaction between BCL-2 and BECLIN1 [37]. In contrast, the PINK1/Parkin pathway utilizes ubiquitin and adaptor proteins, such as p62, to tether the damaged mitochondrion to the autophagosome [5]. Exactly what physiological conditions activate the two different mitophagy pathways are currently unclear and under intense investigation. The functional difference between the two mitophagy pathways is also unclear. BNIP3 can promote clearance of mitochondria with intact mitochondrial membrane potential [23], whereas PINK1/Parkin-mediated clearance seems to require a loss in mitochondrial membrane potential [38]. Studies have shown that both BNIP3/NIX- and PINK1/Parkin-mediated mitophagy are involved in the normal turnover of mitochondria in the myocardium [16, 39, 40]. Thus, the redundancy of mitophagy pathways ensures the removal of dysfunctional mitochondrial in myocytes. Studies have also found that the PINK1/Parkin pathway is important in adapting to acute stress by clearing damaged mitochondria in myocytes [17, 41]. However, whether BNIP3 and/or NIX plays an important role in clearing mitochondria in response to stress is currently unclear and needs to be investigated.

Interestingly, there is evidence that Parkin coordinates with autophagy receptors to induce mitophagy. For instance, we previously found that BNIP3-mediated autophagy is reduced in Parkin-deficient myocytes [22]. Similarly, the mitophagy receptor NIX is involved in recruiting Parkin to depolarized mitochondria after treatment with CCCP [42]. In this study, we found that, in contrast to Parkin-/- myocytes, activation of BNIP3-mediated autophagy and mitophagy are independent of PINK1. This suggests that BNIP3 coordinates with Parkin, but not PINK1, to induce mitophagy.

Impaired mitochondrial quality control in myocytes will have severe consequences for the heart including accumulation of damaged mitochondria and loss of myocytes. It also reduces the capability of the myocytes to adapt to stress such as myocardial infarction [17] or chronic pressure overload [43]. Clearly, the role of the PINK1/Parkin pathway in mitochondrial quality control is more complex than initially believed, and our understanding is still being refined. However, additional investigations into how the PINK1/Parkin pathway regulate mitochondrial quality control at both the protein and organelle levels and how it coordinates with other mitophagy pathways are needed to further our understanding and help identify whether this pathway can be targeted therapeutically to preserve mitochondrial function in myocytes.
